# Molecular-based estimate of species number, phylogenetic relationships and divergence times for the genus *Stenotaenia* (Chilopoda, Geophilomorpha) in the Italian region

**DOI:** 10.3897/zookeys.510.8808

**Published:** 2015-06-30

**Authors:** Laura Del Latte, Francesca Bortolin, Omar Rota-Stabelli, Giuseppe Fusco, Lucio Bonato

**Affiliations:** 1 Department of Biology, University of Padova, via U. Bassi 58/B, 35131 Padova, Italy; 2 Department of Sustainable Agro-Ecosystems and Bioresources, Edmund Mach Foundation, San Michele all’Adige, 38010 Trento, Italy

**Keywords:** COI, DNA barcoding, evolution, genetic distances, molecular dating, 28S

## Abstract

*Stenotaenia* is one of the largest and most widespread genera of geophilid centipedes in the Western Palearctic, with a very uniform morphology and about fifteen species provisionally recognized. For a better understanding of *Stenotaenia* species-level taxonomy, we have explored the possibility of using molecular data. As a preliminary assay, we sampled twelve populations, mainly from the Italian region, and analyzed partial sequences of the two genes *COI* and *28S*. We employed a DNA-barcoding approach, complemented by a phylogenetic analysis coupled with divergence time estimation. Assuming a barcoding gap of 10–16% K2P pairwise distances, we found evidence for the presence of at least six *Stenotaenia* species in the Italian region, which started diverging about 50 million years ago, only partially matching with previously recognized species. We found that small-sized oligopodous species belong to a single clade that originated about 33 million years ago, and obtained some preliminary evidence of the related genus *Tuoba* being nested within *Stenotaenia*.

## Introduction

*Stenotaenia* Koch, 1847 is one of the largest and most widespread genera of geophilid centipedes occurring in the Western Palearctic. Species of *Stenotaenia* have been recorded mainly from the Italian region, through the Balkan peninsula and the Aegean islands, to Anatolia (Bonato and Minelli 2008). However, there are also sparse records reaching the British isles, to the west (Barber 2009), the Alborz mountains in Iran (R. Zarei, unpublished data), to the east, and the Atlas chain plus Crete, Cyprus and Israel, to the south (Chipman et al. 2013, Simaiakis et al. 2013).

Like in most other centipedes, taxonomic recognition and delimitation of species in *Stenotaenia* have so far been based on scanty morphological evidence. However, all *Stenotaenia* species are remarkably similar in body anatomy and several fine morphological details, including most of the characters that are traditionally considered diagnostic at the species level in other geophilid genera (e.g., details of the labrum and the maxillary complex, shape and denticulation of the forcipules, arrangement of the sternal pore areas along the trunk, structure and shape of the legs of the ultimate pair and the associated metasternite, and arrangement of the coxal pores). On the contrary, *Stenotaenia* exhibits high variability in the number of trunk segments and adult body size, so that species-level current taxonomy is based almost exclusively on these two characters. Extreme morphologies are represented by *Stenotaenia romana* (Silvestri, 1895), which is reported being less than 17 mm long when fully grown, with some specimens having only 43 leg-bearing segments, and *Stenotaenia sturanyi* (Attems, 1903), reaching 77 mm in length, with specimens having up to 115 leg-bearing segments (Bonato and Minelli 2008). According to a recent critical reassessment of all available morphological information, no more than fifteen species have been recognized in *Stenotaenia* and more than half of these should be considered valid only provisionally, because supporting evidence is inadequate (Bonato and Minelli 2008).

For a better understanding of *Stenotaenia* species-level taxonomy, we have explored the possibility of using molecular data. As a preliminary assay, we analyzed two genes in a sample of populations from a significant part of the geographic range of the genus. Our aim was to estimate how many species could be recognized on the basis of DNA sequences, especially in comparison with the taxonomic scheme currently in use. We adopted a DNA-barcoding approach, complemented by a phylogenetic analysis coupled with divergence time estimation.

## Material and methods

### Sampling and DNA extraction

The target of our study was the genus *Stenotaenia* according to the taxonomic concept and circumscription currently in use (Bonato and Minelli 2008).

**Figure 1.**
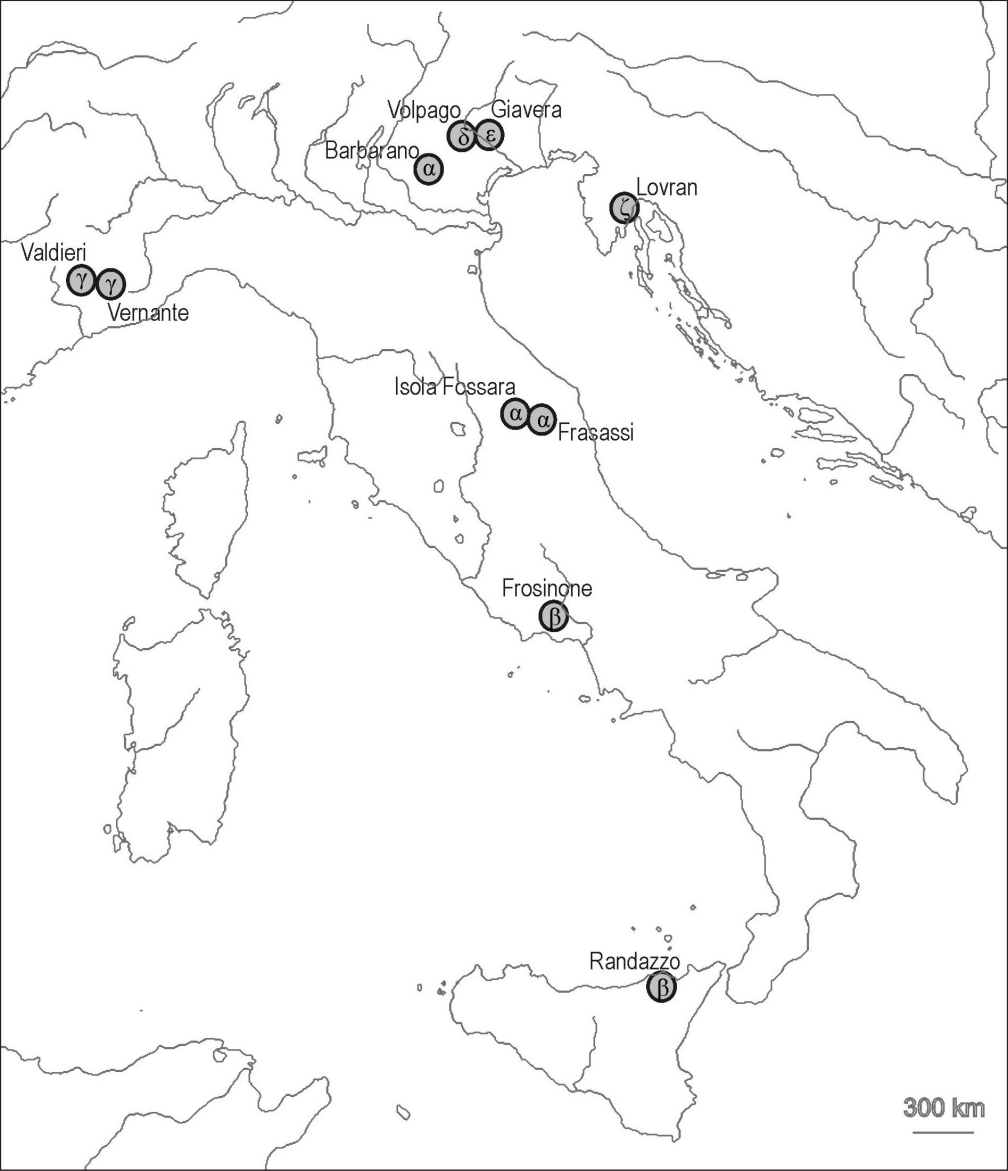
Sampling localities of *Stenotaenia* in the Italian region. Greek letters refer to the species tentatively recognized after the analyses (see text).

Our sampling focused on the western part of the known range of the genus. This area is centred on the Italian region s.l., which extends from the Alps and Istria, through the entire Italian peninsula, to the Italian islands (Fig. 1). So far, this area has been investigated much more intensively than the remaining range of the genus (Bonato and Minelli 2008) and it represents the only part of the range for which a consistent taxonomic scheme for *Stenotaenia* has been developed. We sampled ten specimens from ten different populations within this area and two specimens from localities at the eastern borders of the known range of the genus, i.e. from Cyprus and from Iran (Table 1). Most of the specimens were obtained directly by recent sampling in the field, because most of the material already available in collections has proved unsuitable for DNA extraction, amplification and sequencing; in fact, the DNA of materials stocked in ethanol 70% or in other solutions used for morphological studies (i.e., lactophenol) easily degrades.

**Table 1.** Sampled specimens of *Stenotaenia*, arranged west to east and then north to south. The preliminary identification is just a tentative one, based only on the few morphological characters hitherto proposed as diagnostic at the species-level, including number of legs and geographical provenance (Bonato and Minelli 2008). Abbreviations for collectors: FB = F. Bortolin, GF = G. Fusco, LB = L. Bonato, MZ = M. Zapparoli, RZ = R. Zarei. Abbreviations for repositories of voucher specimens: BM = Bonato-Minelli’s collection, Department of Biology, University of Padova; TE = Zoological Museum, University of Tehran.

Label	Region	Locality	Date and collectors	# of leg pairs	Sex	Repository and code	Species identification	
							preliminary	post analyses
Valdieri	Maritime Alps	near Valdieri: Sant’Anna di Valdieri	24.IX.2012. FB, GF, LB lg	61	♂	BM 3876	Stenotaenia cf. sorrentina	species γ
Vernante	Maritime Alps	near Vernante: Valle Grande	23.IX.2012. FB, GF, LB lg	63	♀	BM 3830	Stenotaenia cf. sorrentina	species γ
Barbarano	Berici hills	near Barbarano: San Giovanni	22.X.2012. LB lg	49	♀	BM 3570	*Stenotaenia romana*	*Stenotaenia romana*
Volpago	Venetian Prealps	near Volpago del Montello: Valle Padovana	2010. LB lg	63	♀	BM 767	Stenotaenia cf. sorrentina	species δ
Giavera	Venetian Prealps	near Giavera del Montello: Valle del Cavalletto	2013. FB, GF, LB lg	77	♂	BM 1787	*Stenotaenia linearis*	species ε
Lovran	Istria	near Lovran: Lovranska Draga-Visoče	23.IX.2011. LB lg	55	♂	BM 1816	*Stenotaenia palpiger*	*Stenotaenia palpiger*
Isola Fossara	Umbro-Marchigian Apennines	near Isola Fossara: Costa del Corno	2.XI.2007. LB lg	57	♀	BM 601	*Stenotaenia sorrentina*	*Stenotaenia romana*
Frasassi	Umbro-Marchigian Apennines	near Frasassi	XII.2010. LB lg	49	♀	BM 1453	*Stenotaenia romana*	*Stenotaenia romana*
Frosinone	Ausoni hills	near Frosinone: Falvaterra-Pastena	8.XII.2011. MZ lg.	63	-	BM 3668	*Stenotaenia sorrentina*	*Stenotaenia sorrentina*
Randazzo	Sicily	near Randazzo: Bosco del Flascio	8.IV.2013. FB, GF, LB, RZ lg	61	-	BM 4553	*Stenotaenia sorrentina*	*Stenotaenia sorrentina*
Cyprus	Cyprus	near Neo Chorio: Smigies-Kefalovrysia	2.I.2010. LB lg	75	♂	BM 1478	*Stenotaenia naxia*	*Stenotaenia naxia*
Iran	Alborz Mountains	near Dasht-e Lar	23.V.2012. RZ lg	-	-	TE 4298	*Stenotaenia* sp.	*Stenotaenia* sp.

After a preliminary species identification based on morphology (Table 1) following Bonato and Minelli (2008), the specimens were fixed in absolute ethanol to preserve DNA from degradation. Total genomic DNA was extracted from a dissected intermediate portion of the trunk of each specimen, using the DNeasy Blood and Tissue kit (Qiagen, Hilden, Germany).

### DNA amplification and sequencing

We sequenced a portion of the *cytochrome c oxidase subunit I* (*COI*) using the primer pair LCOI490/HCO2198 (Folmer et al. 1994), which amplifies an approximately 700 bp long fragment, and a portion of *28S rRNA* (*28S*) using the primer pair 28SD1F/28SrD4b (Boyer and Giribet 2007, Edgecombe and Giribet 2006), which amplifies a 1050 bp long fragment. For *COI* amplification we employed the following PCR conditions: an initial denaturation step at 95 °C for 5 min, followed by a variable number of cycles (27–35) including denaturation at 94 °C for 1 min, annealing (ranging from 40 to 47 °C) for 1 min and extension at 72 °C for 1.5 min, then a final step at 72 °C for 7 min. For *28S* amplification we applied the same thermal cycling profile except for the annealing step, ranging from 42 to 50 °C for 1 min. Each PCR product was screened for the potential successful amplification by electrophoresis on 1% agarose gel in 1X TAE and purified using MinElute PCR Purification Kit (Qiagen). Then it was directly sequenced on both strands with the same primer sets as used for amplification, using an ABI 3130 XL automatic capillary sequencer (Applied Biosystems, Branchburg, USA; service provided by BMR Genomics, Padova, Italy).

We obtained *COI* sequences for all 12 specimens of *Stenotaenia* (between 642 bp and 647 bp long) and *28S* sequences from 11 *Stenotaenia* specimens (between 952 and 1005 bp long) (Table 2).

**Table 2.** GenBank accession numbers, GC-skew and GC-content of the sequences of all specimens of *Stenotaenia* and the outgroup species included in the phylogenetic analysis.

Label	Repository and code	GenBank accession number		GC-skew		GC-content (%)	
		*COI*	*28S*	*COI*	*28S*	*COI*	*28S*
Valdieri	BM 3876	LN811344	LN810434	-0.189	0.051	40.0	61.0
Vernante	BM 3830	LN811343	LN810433	-0.183	0.053	39.7	60.9
Barbarano	BM 3570	LN811341	LN810431	-0.176	0.054	38.2	59.3
Volpago	BM 767	LN811336	-	-0.246	-	45.9	-
Giavera	BM 1787	LN811339	LN810429	-0.136	0.044	38.6	61.5
Lovran	BM 1816	LN811340	LN810430	-0.201	0.051	41.4	57.5
Isola Fossara	BM 601	KF569300	KF569278	-0.237	0.047	43.0	59.3
Frasassi	BM 1453	LN811337	LN810437	-0.244	0.054	42.5	59.8
Frosinone	BM 3668	LN811342	LN810432	-0.244	0.058	41.4	61.1
Randazzo	BM 4553	LN811346	LN810435	-0.206	0.056	40.6	61.5
Cyprus	BM 1478	LN811338	LN810428	-0.188	0.069	46.8	61.8
Iran	TE 4298	LN811345	LN810436	-0.218	0.040	43.9	62.1
*Arctogeophilus glacialis*	-	KF569291	KF569268	-0.254	0.049	46.9	55.7
*Clinopodes carinthiacus*	-	KF569292	KF569269	-0.156	0.068	41.1	61.0
*Geophilus alpinus*	-	KF569294	KF569271	-0.235	0.080	47.3	57.5
*Geophilus electricus*	-	AY288750	HM453296	-0.217	0.072	43.4	58.1
*Geophilus flavus*	-	KF569296	KF569273	-0.232	0.063	42.4	61.5
*Tuoba sydneyensis*	-	AY288751	HM453297	-0.205	0.055	39.3	59.7

Finch TV 1.4.0 (Geospiza, PerkinElmer) was used to check each chromatogram for nucleotide signal intensity and whole sequence signal strength. Sequences were edited manually to obtain a more accurate reading. For each specimen, forward and reverse sequences of the gene were aligned with default parameters with Clustal W2 (Larkin et al. 2007) and combined in a single sequence. After that, datasets of *COI* and *28S* of *Stenotaenia* specimens were aligned with the same procedure.

### Analyses of sequence variation

For delimiting species in our sample through a DNA-barcoding approach, we calculated the pairwise distances of the *COI* sequences between all 12 *Stenotaenia* specimens in two alternative ways, namely by K2P distances (which is a standard for DNA-barcoding; e.g., Hebert et al. 2003, Ratnasingham and Hebert 2007, Chevasco et al. 2014) and p-distances (which is also used in arthropods; e.g., Montagna et al. 2013), treating gaps with partial deletion and estimating standard errors by 500 bootstrap pseudoreplicates. The software MEGA 6 (Tamura et al. 2013) was employed. We analyzed the frequency distribution of both kinds of pairwise distances in order to recognize the putative *barcoding gap*. This is the interval which is expected to separate within-species distances from between-species distances in the distribution of pairwise distances (Hebert et al. 2003, Ratnasingham and Hebert 2007).

### Phylogenetic analysis

For the phylogenetic analysis of the *Stenotaenia* sequences, we chose as outgroups six species of Geophilidae (Table 2) for which sequences of *COI* and *28S* were already available. These species were selected as representative of the genera reputed to be the most strictly related to *Stenotaenia* according to previous phylogenetic analyses (Murienne et al. 2010, Bonato et al. 2014) and, as far as possible, including the type species of these genera. Because compositional biases may interfere with the phylogenetic signal of the dataset (Rota-Stabelli and Telford 2008), we checked whether the outgroup sequences were ingroup-like in the GC-content and the GC-skew.

The full set of ingroup and outgroup sequences were aligned using Clustal W2 (with default parameters) and only the positions shared by all sequences were considered for the analyses. The single genes and the concatenated sequences were analyzed by a maximum likelihood (ML) approach with PHYML 3.1 (Guidon et al. 2010) and by a Bayesian approach using BEAST v1.7.2 (Drummond et al. 2012). We estimated the best-fitting replacement model according to the Akaike information criterion (Posada and Buckley 2004) implemented in jModelTest v2.1.1 (Darriba et al. 2012). The ML analyses were bootstrapped using 100 pseudoreplicates. Newick output trees were visualized and manually rooted (assuming, as far as possible, the monophyly of the ingroup) with FigTree v1.4.0 (Rambaut 2009).

### Molecular dating

To estimate divergence dates between *Stenotaenia* sequences, we used the Bayesian method implemented in BEAST v1.7.2 with XML input files prepared using BEAUti v1.7.2 (Drummond et al. 2012). Since no fossil record exists for any of the taxa represented in our dataset, we relied on both a root prior and a replacement rate derived from previous date estimates. As root prior we used an estimate of 200 million years (Ma), with a permissive SD=50, for the basal split in the phylogeny obtained, between the most basal outgroup and the remaining taxa, according to the dated phylogeny of Murienne et al. (2010). As replacement rate we assumed a value of 0.0016 substitutions/site/Ma, with SD = 0.0010, which was previously estimated analyzing a *18S+28S* dataset from a wide range of arthropods, including myriapods (Rota-Stabelli et al. 2013). The choice of this rate was motivated by a lack of more taxon-specific rate for centipede *28S*. We modelled the molecular replacement with the best-fitting model selected by the Akaike information criterion, and the rate distribution using a random clock model. All other priors were those of BEAST at default settings.

## Results

### Pattern of sequence diversity

**Figure 2.**
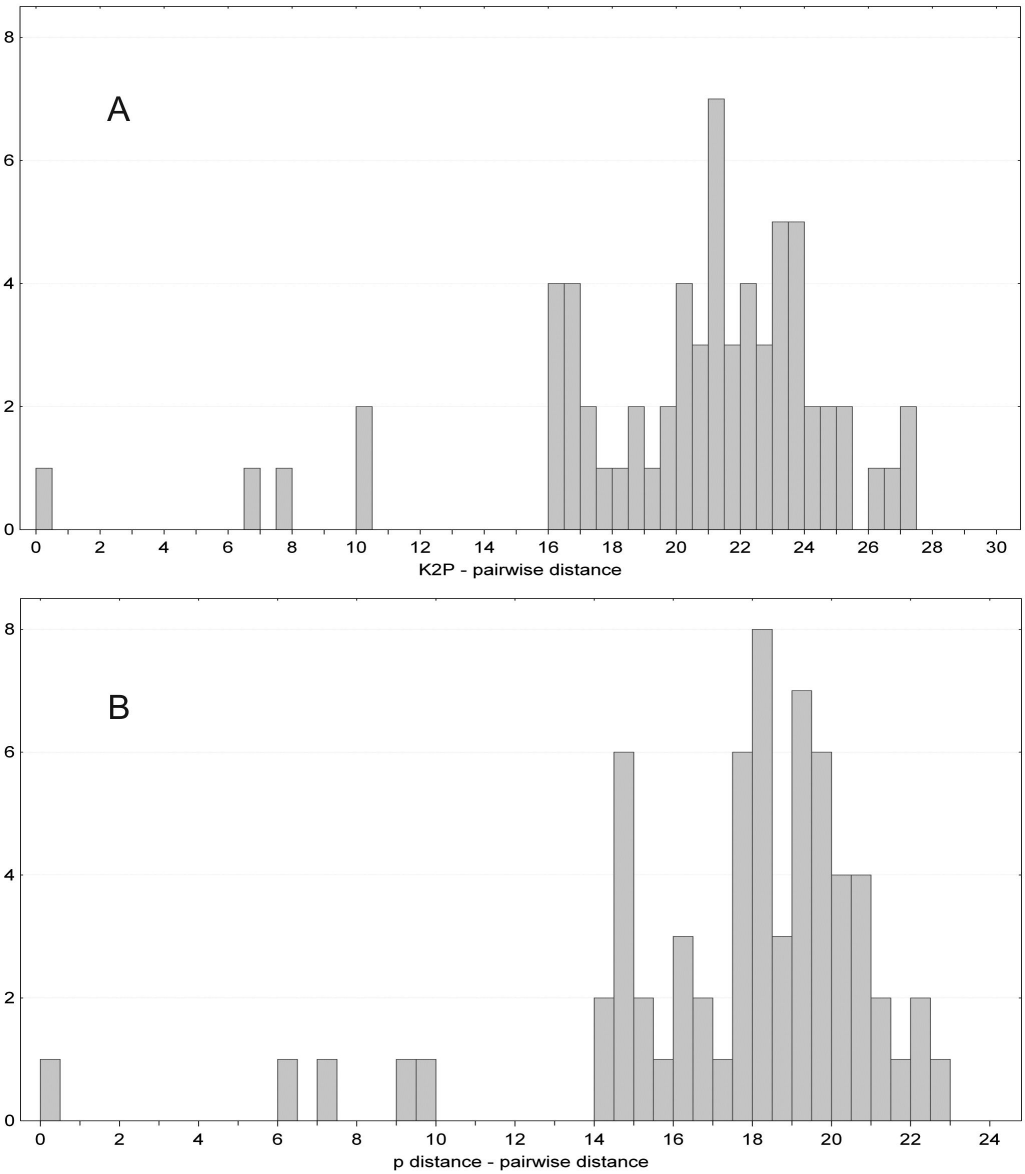
Frequency distribution of *COI* pairwise distances. **A** K2P distances. **B** p-distances.

Considering the pairwise distances of *COI* between the sampled specimens of *Stenotaenia* (Table 3), the minimum distance (about 0.5% for both the K2P and the p-distances) was recorded between two specimens from the Western Alps (Valdieri, Vernante), which had been previously recognized as belonging to a single species based on morphology (Table 1). The maximum distance (27.4% for the K2P distances, 22.6% for the p-distances) was estimated between a specimen from Cyprus and a specimen from the Eastern Alps (Barbarano), previously recognized as belonging to distinct species based on morphology (Table 1). Considering the frequency distributions of the pairwise distances of both models (Fig. 2), two major intervals of zero frequency might be recognized: a lower one (0.5–6.5% for K2P, 0.5–6.2% for p-distances) and a higher one (10.4–16.1% for K2P, 9.5–14.4% for p-distances).

**Table 3.** Pairwise distances (in percentage, with standard errors in parenthesis) between all *Stenotaenia* specimens, based on *COI* sequences, obtained with the K2P model (top right) and with the p-distances (bottom left).

	Cyprus	Volpago	Lovran	Barbarano	Frosinone	Isola Fossara	Frasassi	Randazzo	Iran	Vernante	Valdieri	Giavera
Cyprus		26.6 (2.3)	27.0 (2.2)	27.4 (2.3)	25.1 (2.1)	26.0 (2.2)	25.3 (2.1)	24.5 (2.1)	23.4 (2.0)	24.4 (2.0)	24.4 (2.0)	22.9 (2.0)
Volpago	22.1 (1.5)		22.4 (1.9)	20.5 (1.8)	16.6 (1.7)	21.2 (1.8)	21.2 (1.8)	16.6 (1.6)	19.9 (1.9)	18.9 (1.9)	19.5 (2.0)	23.8 (2.0)
Lovran	22.4 (1.5)	19.2 (1.4)		19.5 (1.8)	23.7 (2.0)	23.0 (2.0)	21.7 (1.9)	23.5 (2.0)	22.6 (1.9)	22.4 (1.8)	23.0 (1.9)	23.0 (1.9)
Barbarano	22.6 (1.5)	17.8 (1.4)	17.0 (1.4)		18.0 (0.17)	10.4 (1.2)	10.2 (1.2)	20.3 (1.8)	21.3 (1.9)	20.4 (1.8)	21.0 (1.9)	24.0 (2.0)
Frosinone	21.2 (1.5)	14.7 (1.3)	20.1 (1.4)	15.9 (1.4)		18.5 (1.7)	18.7 (1.6)	6.5 (1.1)	16.1 (1.6)	17.2 (1.7)	17.4 (1.8)	23.5 (2.0)
Isola Fossara	21.6 (1.5)	18.2 (1.3)	19.5 (1.4)	9.5 (1.0)	16.3 (1.3)		7.6 (1.1)	20.6 (1.9)	22.3 (2.0)	21.3 (1.9)	21.6 (2.0)	23.8 (1.9)
Frasassi	21.2 (1.5)	18.2 (1.3)	18.6 (1.4)	9.5 (1.1)	16.4 (1.3)	7.0 (1.0)		21.0 (1.8)	20.1 (1.8)	20.2 (1.8)	20.5 (1.8)	23.1 (1.9)
Randazzo	20.7 (1.4)	14.7 (1.3)	20.0 (1.4)	17.6 (1.4)	6.2 (0.9)	17.8 (1.3)	18.1 (1.3)		16.5 (1.6)	16.8 (1.8)	16.6 (1.8)	22.3 (1.9)
Iran	19.9 (1.5)	17.2 (1.4)	19.3 (1.4)	18.4 (1.4)	14.4 (1.3)	19.0 (1.4)	18.1 (1.4)	14.6 (1.2)		16.2 (1.6)	16.4 (1.6)	24.7 (2.0)
Vernante	20.6 (1.4)	16.2 (1.4)	19.2 (1.4)	17.8 (1.4)	15.2 (1.4)	18.4 (1.4)	17.6 (1.4)	14.9 (1.4)	14.4 (1.3)		0.5 (0.3)	21.3 (1.9)
Valdieri	20.6 (1.4)	16.7 (1.4)	19.6 (1.4)	18.2 (1.4)	15.3 (1.4)	18.6 (1.3)	17.8 (1.4)	14.7 (1.4)	14.5 (1.3)	0.5 (0.3)		21.9 (1.9)
Giavera	19.5 (1.4)	20.1 (1.4)	19.6 (1.4)	20.3 (1.4)	19.9 (1.4)	20.1 (1.3)	19.6 (1.3)	19.0 (1.4)	20.7 (1.4)	18.2 (1.3)	18.7 (1.3)	

Assuming a barcoding gap corresponding to the lower observed gap, we would obtain 11 species of *Stenotaenia* from our sample of 12 specimens, of which nine species in the Italian region (between Alps, Istria and Sicily), with only two specimens from the Western Alps resolved as conspecific. On the contrary, assuming a barcoding gap corresponding to the higher observed gap, we would obtain eight species of *Stenotaenia*, of which six in the Italian region.

### Phylogenetic relationships

After the alignment of ingroup and outgroup sequences, the average nucleotide composition of *COI* turned to be A = 0.289, C = 0.248, G = 0.163, T = 0.299, and that of *28S* resulted as A = 0.219, C = 0.283, G = 0.317, T = 0.181. A bias against G-C in the composition of *COI* has been observed in other Chilopoda as well (Spelda et al. 2011, Oeyen et al. 2014). The GC-contents (Table 2) of the sequences of the outgroups turned out being within the range of variation of *Stenotaenia* sequences (38.2–46.8% for *COI*, 57.5–62.1% for *28S*), with minor exceptions for *Arctogeophilus glacialis* and *Geophilus alpinus* (slightly higher for *COI*, slightly lower for *28S*). Also the GC-skew values (Table 2) of most of the outgroup sequences turned out within the range of variation of the *Stenotaenia* sequences (between -0.246 and -0.136 for *COI*, between 0.040 and 0.069 for *28S*), but with some exceptions (slightly lower for *COI* in *Arctogeophilus glacialis*, higher for *28S* in *Geophilus alpinus* and *Geophilus electricus*).

For the ML phylogenetic analysis, the Generalized Time Reversible model with proportion of invariable sites and a Gamma distribution (GTR+I+G) with four discrete categories was selected as the best-fit model for nucleotide substitution with the Akaike information criterion. We applied this model for the datasets of the *COI* sequences and the *28S* sequences, when analyzed separately and when concatenated.

**Figure 3.**
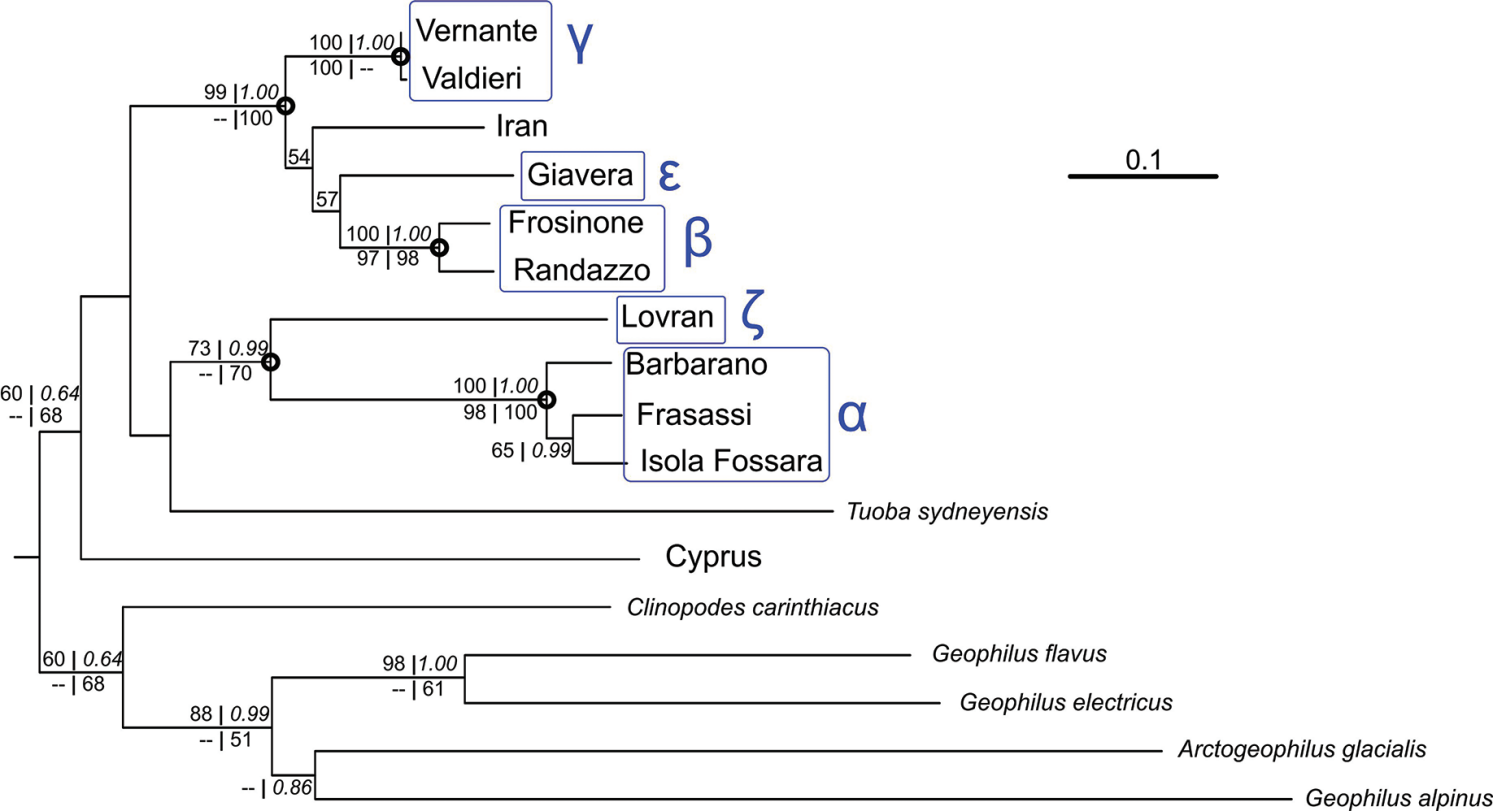
Maximum likelihood phylogeny. ML tree obtained from concatenated *COI* and *28S* sequences, by the GTR+I+G model, and manually rooted. The following support values are indicated at the nodes (only for those present in the topology obtained from the concatenated sequences): ML bootstrap for the analysis of concatenated genes (upper left); Bayesian posterior probabilities (upper right, in italics); ML bootstrap for the analysis of *COI* sequences (lower left); ML bootstrap for the analysis of *28S* sequences (lower right). Bootstrap values < 50% and posterior probabilities < 0.50 are not shown. Circles indicate ingroup nodes that are highly supported in the tree based on concatenated sequences. Terminal node groupings indicated by Greek letters refer to the species tentatively recognized (see text and Fig. 1). The specimen from Volpago (species δ) is absent because its *28S* sequence was not obtained.

The ML tree, obtained from the concatenated sequences of the 11 *Stenotaenia* specimens from which we got workable sequences for both genes, is shown in Fig. 3. Very similar topologies for the ingroup were found in the ML trees obtained from the single genes separately and in the Bayesian tree obtained from the concatenated sequences (trees not shown, but node supports shown on the ML tree, Fig. 3). Three groups of *Stenotaenia* specimens were found to be strongly supported (at least when analyzing the two genes together) and corresponded to groups obtained by the DNA-barcoding analysis assuming the higher barcoding gap (10.4–16.1% for K2P, 9.5–14.4% for p-distances). These are: a group including three specimens between the Eastern Prealps and the northern part of the Italian peninsula (Barbarano, Frasassi, Isola Fossara), a second group with the two specimens from the southern part of the Italian peninsula and Sicily (Frosinone, Randazzo), and a last group with both specimens from the Western Alps (Vernante, Valdieri). In addition, we found strong support for a clade including the first group listed above and a specimen from Istria (Lovran), and for another clade including the other two groups already listed plus another specimen from the Eastern Prealps (Giavera) and a specimen from Iran.

However, the monophyly of the genus *Stenotaenia*, as currently circumscribed, was not recovered in our analyses: the specimen representative of the genus *Tuoba* was found nested within the clade encompassing all *Stenotaenia* specimens in both ML and Bayesian analyses, and also in the ML analysis of *28S*, even though in different positions (Fig. 3); in the ML analysis of *COI*, instead, the specimen representative of the genus *Clinopodes* was nested within *Stenotaenia*.

### Divergence time

**Figure 4.**
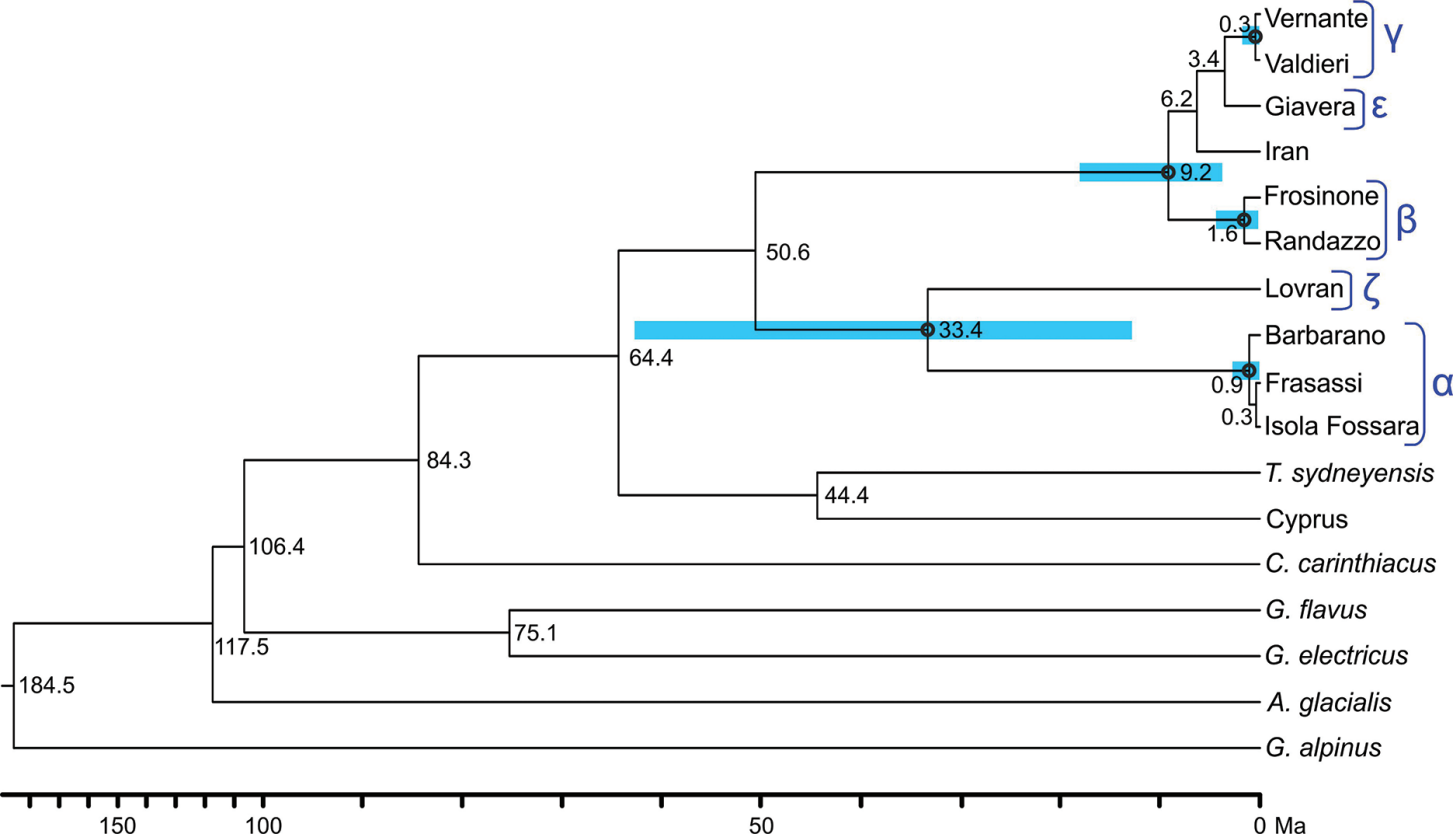
Dated phylogeny. Estimates of divergence time, calculated using *28S* sequences and two priors (age of the root and substitution rate) in the package BEAST v1.7.2 (see text). 95% High Posterior Density intervals are represented by coloured bars for the most robust nodes, emphasized by a circle. Greek letters refer to the species tentatively recognised (see Fig. 1). The tree has the same topology of the concatenated ML tree of Fig. 3, but for the position of *Tuoba sydneyensis* and the relationships within the group formed by species β, ε, γ and the specimen from Iran. The specimen from Volpago (species δ) is absent because its *28S* sequence was not obtained. Time scale is different in the two intervals 0–100 and 100–200 Ma.

By applying a molecular clock on a Bayesian analysis of the *28S* sequences, we obtained a tree topology (Fig. 4) largely consistent with ML and Bayesian trees obtained from the concatenated dataset (Fig. 3). Differences are limited to the unstable position of *Tuoba* and two nodes with weak support.

The common ancestor of all *Stenotaenia* representatives (including *Tuoba* if it comprises a monophyletic group together with *Stenotaenia*) was dated at about 64 Ma. The divergence between the two major groups of *Stenotaenia* present in the Italian region (one represented by specimens from the northern part of the Italian peninsula, besides from the Eastern Prealps and Istria; another represented by specimens from the southern part of Italian peninsula and Sicily, besides from the Western Alps and the Eastern Prealps) was dated at about 51 Ma. Within the first group, the lineage of the specimen from Istria diverged from the remaining lineages around 33 Ma. The estimated age of the subsequent divergences are all younger than 10 Ma. In particular, for the groups obtained by the DNA-barcoding analysis of *COI* sequences assuming a higher barcoding gap and well supported in the phylogenetic analyses (see above), the common ancestor of each group was dated younger than 1.6 Ma, whereas all divergences between these groups and other specimens of *Stenotaenia* were all dated older than 3.4 Ma.

### Putative species of *Stenotaenia*

The results of the DNA-barcoding analysis of *COI* sequences, of the phylogenetic reconstructions on the basis of the two genes, and of the divergence date estimation based on the *28S* sequences, all agree in suggesting that at least six species of *Stenotaenia* are recognizable in our sample from the Italian region (Fig. 1): α (three populations between the Eastern Prealps and the northern part of the Italian peninsula), β (two populations from the southern part of Italian peninsula and Sicily), γ (two populations from Western Alps), δ and ε (each represented by a single population in the Prealps), ζ (one population from Istria). Additionally, the two populations from Cyprus and Iran probably belong to different species.

## Discussion

The analysis of the pairwise distances of the *COI* sequences, following our DNA-barcoding approach, suggested two alternative putative barcoding gaps, both consistent with the results of our phylogenetic and dating analyses. However, the two gaps entail different scenarios for the cladogenesis in *Stenotaenia*, including a different estimate of the minimum number of species inhabiting the Italian region.

Assuming a low barcoding gap (1–6% of K2P distances) would be in agreement with a threshold around 1% between intra- and inter-specific distances, as more frequently found and applied in a large assortment of animal taxa, including vertebrates and some groups of arthropods (Ratnasingham and Hebert 2007, Chevasco et al. 2014). However, in this case we should conclude that (i) almost every single specimen in our small sample would actually represent a distinct species, (ii) some of these species separated as recently as a few hundred thousand years ago, and (iii) at least nine species of *Stenotaenia* actually inhabit the Italian region. In this scenario, a remarkable number of species would be expected for the entire geographic range of the genus, in face of the morphological uniformity between species and departing dramatically from the taxonomic scheme in use (Bonato and Minelli 2008).

As an alternative, assuming a higher barcoding gap (10–16% of K2P distances) would be in agreement with gaps estimated in other centipedes, in the very few studies so far published: a gap in p-distances between 9% and 14% for the scolopendromorph genus *Scolopendra* (Oeyen et al. 2014), a gap in K2P distances between 7% and 10% for the lithobiomorph genus *Eupolybothrus* (Stoev et al. 2010, 2013) and average intraspecific vs. interspecific K2P distances of 7% vs. 18%, for a centipede sample from the Bavarian fauna (Spelda et al. 2011). This is also in agreement with the divergence time analysis, as under the higher barcoding gap scenario all interspecific nodes would be older than one Ma, which is a more reasonable figure (Coyne and Orr 2004). In this scenario, we should acknowledge a lower number of species in our sample. On the basis of this more parsimonious assumption, the minimum number of six species we obtain for the Italian region is nonetheless higher than the four species hitherto recognized on the basis of morphology, a number which besides is derived by a far more extensive geographic sampling (Bonato and Minelli 2008).

Despite evidence being very preliminary, species delimitation suggested by our study may be tentatively compared and matched with these four morphospecies.

The putative species α might correspond to *Stenotaenia romana* (Silvestri, 1895), even if it includes also a specimen initially identified as *Stenotaenia sorrentina*. *Stenotaenia romana* was customarily distinguished from all other species in the Italian region by its remarkably minute body size (total length not surpassing 17 mm) and a distinctly lower range of variation in the number of trunk segments (43–49 pairs of legs, Bonato and Minelli 2008). *Stenotaenia romana* inhabits mainly the Tyrrhenian side of the Italian peninsula, from Liguria to Campania, including some minor islands (e.g., Ischia and Elba), and also Sardinia; apparently disjunct populations are present in the Euganei and Berici hills (unpublished data).

Species β confidently corresponds to *Stenotaenia sorrentina* (Attems, 1903), which is usually circumscribed by having intermediate body size (at least 20 mm when fully grown) and an intermediate number of trunk segments (usually 53–67 leg pairs, Bonato and Minelli 2008). This morphospecies has been frequently recorded in the Italian peninsula from Maritime and Ligurian Alps to Gargano and Calabria, and more rarely and doubtfully also from the Prealps. It is also reported from several islands, including Sardinia and minor Tyrrhenian islands (e.g., Elba, Ponza, Capri and Ischia). Our sample from Sicily (Table 1) confirms the presence of this species in this island, from where only old and dubious records were available so far (Bonato and Minelli 2008).

The species ζ may correspond to *Stenotaenia palpiger* (Attems, 1903), which was known so far only for the holotype (total length 15 mm and 49 leg pairs), collected from Istria, about 30 km from the locality of our sample. The species name *palpiger* has been most often ignored and only recently resurrected as a potentially valid species (Bonato and Minelli 2008). However, the identity and distribution of *Stenotaenia palpiger* remain to be clarified, especially with respect to other nominal species of *Stenotaenia* reported from the Dinaric region, including *Stenotaenia antecribellata* (Verhoeff, 1898) and *Stenotaenia cribelliger* (Verhoeff, 1898) (Bonato and Minelli 2008).

The remaining three putative species (γ from Western Alps, δ and ε from Eastern Prealps) cannot be assigned confidently to known species. Different number of legs in their specimens had initially suggested different tentative identification (see Table 1). However, according to their origins, it is impossible to tell which of them, if any, actually belongs to *Stenotaenia linearis*, which is the fourth species traditionally reported for the Italian region. The type locality of *Stenotaenia linearis* is in Baviera (near Regensburg) and based on morphology the species has been reported also from the Alps, as far south as the Maritime Alps (Bonato and Minelli 2008).

While the specimen from Cyprus is confidently recognizable as a different species, *Stenotaenia naxia* (Verhoff, 1901) (Simaiakis et al. 2013), which is also recorded from the Aegean area (Bonato and Minelli 2008), nothing can be said for the specimen from Iran. The number of leg-bearing segments and the morphology of the ano-genital region are unknown because the posterior part of the trunk is missing. This makes it very difficult to associate this specimen to any known species, also because no previous records for the genus are known for this geographic area.

Our preliminary evaluation of the phylogenetic relationships and the divergence dates between these species have implications on the evolutionary history of the group which invite some cautious comments.

All specimens in our sample that are representative of species with smaller body size and lower number of legs (*Stenotaenia romana* and *Stenotaenia palpiger*) apparently represent a single derived clade within the genus, suggesting that these features are synapomorphic for these two species. Considering the entire clade, as far as known, the body length is less than 20 mm long at full growth and the number of leg pairs ranges from 43 (in *Stenotaenia romana*; Bonato and Minelli 2008) to 57 (in the *Stenotaenia romana* specimen from Isola Fossara).

The genus *Tuoba* Chamberlin, 1920, which is currently recognized as distinct from *Stenotaenia* in different respects (morphological features, ecological niche, pattern of distribution; Bonato 2011) turned out to be strictly related to the latter, suggesting also the possibly that *Tuoba* is a derived clade within *Stenotaenia*. The phylogenetic position of *Tuoba sydneyensis* among the *Stenotaenia* representatives in our sample is unstable and weakly supported, and could also be an artefact of our analysis, possibly produced by the attraction among sequences sharing similar GC-content and GC-skew values (Table 2).

This study is a very preliminary attempt to address the diversity of *Stenotaenia* with genetic data. It was limited by a relatively low efficacy in DNA extraction, amplification and sequencing from the available specimens (actually up to 15 specimens from 14 localities had been originally processed). This prevented us from analyzing a larger sample of specimens, and did not permit taking into account variation within populations. Nevertheless, our results could serve as a basis for forthcoming investigations on the genus *Stenotaenia*, not only in the Italian region, but also in the entire distribution range of the genus.
